# Residue Levels of Organochlorine Pesticides in Breast Milk and Its Associations with Cord Blood Thyroid Hormones and the Offspring’s Neurodevelopment

**DOI:** 10.3390/ijerph16081438

**Published:** 2019-04-23

**Authors:** Cheng-Chih Kao, Danielle E. Que, Sayre J. Bongo, Lemmuel L. Tayo, Yi-Hsien Lin, Chun-Wen Lin, Sheng-Lun Lin, Yan-You Gou, Wen-Li Hsu, Cherng-Gueih Shy, Kuo-Lin Huang, Ming-Hsien Tsai, How-Ran Chao

**Affiliations:** 1 Emerging Compounds Research Center, Department of Environmental Science and Engineering, National Pingtung University of Science and Technology, Neipu, Pingtung County 912, Taiwan; 02791@ptch.org.tw (C.-C.K.); power20342000@gmail.com (Y.-Y.G.); hsuwenli0626@gmail.com (W.-L.H.); huangkl@mail.npust.edu.tw (K.-L.H.); 2 Superintendent, Pingtung Christian Hospital, Pingtung City, Pingtung County 900, Taiwan; 3 Department of Environmental Engineering, National Cheng Kung University, Tainan City 701, Taiwan; aryldiazonium@gmail.com; 4 School of Chemical, Biological and Materials Engineering and Sciences, Mapúa University, Muralla St., Intramuros, Manila 1002, Philippines; sayrebongo@gmail.com (S.J.B.); lemueltayo@yahoo.ca (L.L.T.); 5 Department of Plant Medicine, National Pingtung University of Science and Technology, Neipu, Pingtung County 912, Taiwan; yhlin@mail.npust.edu.tw; 6 Department of Child Care, National Pingtung University of Science and Technology, Neipu, Pingtung County 912, Taiwan; cwlin@mail.npust.edu.tw (C.-W.L.); alantsai@mail.npust.edu.tw (M.-H.T.); 7 Department of Civil Engineering and Geomatics, Cheng Shiu University, Kaohsiung 83347, Taiwan; cbmsgml@gmail.com; 8 Center for Environmental Toxin and Emerging-Contaminant Research, Cheng Shiu University, Kaohsiung 83347, Taiwan; 9 Super Micro Mass Research and Technology Center, Cheng Shiu University, Kaohsiung 833, Taiwan; 10 Research Institute for Life Support Innovation, Research Organization for Nano & Life Innovation, Waseda University, 2-2 Wakamatsucho, Shinjuku, Tokyo 162-8480, Japan; 11 Department of Radiology, Pingtung Christian Hospital, Pingtung City, Pingtung County 900, Taiwan; graycgshy@gmail.com; 12 Institute of Food Safety Management, National Pingtung University of Science and Technology, Neipu, Pingtung County 912, Taiwan

**Keywords:** breast milk, thyroid hormones, organochlorine pesticides, Bayley-III, infant neurodevelopment

## Abstract

Previous studies have demonstrated that organochlorine pesticide (OCP) exposure has a negative impact on the neurological function of infants. Only a few reports have investigated the thyroid and growth hormones and their relationship to neurodevelopment after human exposure to OCPs, especially in the case of infants. Our goal was to determine whether breastmilk OCP residues were associated with negative impacts and/or alterations in the neurodevelopment of infants among specific southern Taiwanese mother–breastfed infant pairs. Our subjects (*n* = 55 pairs) were recruited from southern Taiwan between 2007 and 2010. The thyroid and growth hormone levels in the cord blood samples collected after childbirth were determined. The breastmilk was gathered within one month after childbirth for the determination of OCP levels using a high-resolution gas chromatograph with mass spectrometry, and the neurodevelopment of 10–12-month-old infants was examined using the Bayley Scales of Infant and Toddler Development^®^, Third Edition (Bayley-III). It was observed that 4,4′-dichlorodiphenyl-dichloroethylene (4,4′-DDE) (mean = 10.3 ng/g lipid) was the most predominant OCP compound in the breastmilk samples. At higher concentrations (>75th percentile), specific OCPs were associated with significantly lower levels of thyroid and growth hormones than at lower concentrations (<75th percentile). Significantly higher odds ratios (ORs) were observed for binary cognitive (OR = 8.09, *p* = 0.025 for 4,4′-DDT), language (OR = 11.9, *p* = 0.013 for 4,4′-DDT) and social–emotional (OR = 6.06, *p* = 0.01 for trans-CHL) composite scores for specific OCPs belonging to the lower exposure group as compared to the higher OCP exposure group. The five domain Bayley-III infant neurodevelopment outcomes were negatively associated with specific OCPs in the breast milk samples based on the redundancy analysis (RDA) test. Bayley-III scales, which include cognitive, language, motor, social-emotional, and adaptive behavior scales, could be predicted by 4,4′-DDT, endrin, endosulfan I, heptachlor, or heptachlor epoxide using multivariate linear regression models with adjustment for maternal age, pre-pregnant BMI, parity, and infant gender. In conclusion, although our study showed that postnatal exposure to breast milk OCPs may be associated with infant neurodevelopmental outcomes and that prenatal exposure, if extrapolated from breastmilk levels, is associated with changes in thyroid and growth hormones that may have effects on neurodevelopment, these associations are only suggestive; thus, further studies are recommended for confirmation.

## 1. Introduction

Organochlorine pesticides (OCPs) are environmental contaminants that are ubiquitous in the environment of Taiwan despite being banned in Taiwan since 1975 [1,2] due to their reported toxicities in animals and humans. OCPs are classified as a class of persistent organic pollutants (POPs) that accumulate in environmental and biological matrices. Although OCPs are listed in the Stockholm Convention and have been banned in several countries, their usage in other developing countries still continues [1]. OCPs used for agricultural and sanitary purposes released before the ban are still present in the environment and biota [3,4,5,6,7,8]. OCPs are also recognized as endocrine disruptors because they can act as low estrogenic or anti-estrogenic compounds at low exposure levels. Additionally, OCPs bioaccumulate and are biomagnified along the food chain due to their high lipophilicity, high persistence, and long half-lives, thus allowing them to cause adverse health effects in humans, such as endocrine disruption, reproductive toxicity, cancer, and neurological disorders [9,10]. In addition to their presence in the environment, OCPs have also been observed in human specimens, such as human breast milk, which is a noninvasive sample that can be used to evaluate OCP levels in humans [1]. According to our previous report [11], dietary habits (e.g., consumption of cow’s milk and beef) and menstrual characteristics (e.g., menstrual period days) in women of childbearing age are related to breast milk OCP levels.

The residues of OCPs in breast milk can reflect a maternal burden that can be utilized to further examine the associations of OCPs with maternal health and their possible health risks to nursing offspring. Additionally, using breast milk to examine the OCP levels in humans allows for further investigation into the possible association of infant health risks to postnatal exposure via breastfeeding (lactational exposure) [3]. Among other health risks, OCPs in breast milk samples have been found to be associated with developmental effects [12,13] and infertility [14]. Prenatal exposure to OCPs has already been associated with adverse health effects on newborns such as reduced birth weight [5], reduced head circumference [15], and delayed mental and psychomotor development [3]. Determining the impact on the health of infants with postnatal exposure through breastfeeding is very important. Furthermore, several studies supporting the role of OCPs as neurotoxicants [10] showed the importance of evaluating breast milk OCP levels and investigating their association with infant neurodevelopment. One study on postnatal exposure to chlordecone carried out by Boucher et al. [16] reported that chlordecone did not have any associations with the neurodevelopment of 18-month-old infants. Similarly, lactational exposure to 4,4′-dichlorodiphenyltrichloroethane (DDT) and 4,4′-dichlorodiphenyldichloroethylene (DDE) did not seem to cause neurodevelopmental impairment in infants. However, low scores for male infants on the Mullen gross motor scale have been associated with breastmilk DDE [17].

Aside from associated neurodevelopmental toxicity, OCP exposure in general has been observed to be associated with hormone levels in both adults and children. 4,4′-DDE in serum from male adults was found to be associated with an increase in total triiodothyronine (TT3) and free thyroxine (FT4) as well as with a decrease in the secretion of thyroid-stimulating hormone (TSH) [15,18]. In a study conducted by Freire et al. [19], the effects of chronic exposure of children (younger than 15 years old) living in Cidade dos Meninos, Rio de Janeiro, Brazil to OCPs on thyroid hormone levels was investigated between the years 2003 and 2004. All OCPs that were detected in 60% of the children, with the exception of heptachlor and methoxychlor, were found to have an association with increasing serum TT3 concentrations. In a study done in Menorca, Spain by Alvarez-Pedrerol et al. [20], they reported that the exposure of preschool children aged 4 years to organochlorines had negative associations with TT3 levels. OCPs found in cord or maternal serum have also been observed in previous studies to be associated with thyroid and growth hormones found in cord or maternal serum. In one Korean study, Kim et al. [21] observed that OCPs such as DDTs and hexachlorobenzene (HCB) in maternal serum were associated with reduced maternal serum T3 or T4 levels. Also in their other study, Kim et al. [21] reported that Σchlordane (ΣCHL) in cord serum was associated with TSH in cord or bloodspot, while p,p′-DDE was found to be associated with cord serum TT3 and TT4. In addition, neonatal thyroid hormones were also found to have associations with maternal exposure to ΣCHL, ΣDDT, or p,p′-DDE. A Belgian study showed that the cord blood TSH levels in newborn males whose cord blood 4,4′-DDE levels were over the limit of detection (LOD) were significantly lower than the TSH levels of those with below the LOD cord blood 4,4′-DDE levels [22]. Although the findings were not significant, Luo et al. [23] indicated that cord blood free thyroxine (FT4) was marginally and negatively associated with cord serum levels of Σhexachlorcyclohexanes (ΣHCHs), 4,4′-DDE, and methoxychlor. This Chinese study also showed that after adjustment for confounding factors (age of pregnant mothers, education level, monthly household income, parity, and sex of the newborns), increased cord plasma TSH levels were associated with high cord plasma levels of aldrin, dieldrin, ∑dichlorodiphenyltrichloroethanes (ΣDDTs), ΣDrins, and ΣOCPs, and it was concluded that the thyroid hormone levels of newborns may be associated with in utero exposure to certain OCPs.

Several studies have been conducted to determine the associations of breast milk OCPs to infant neurodevelopment; however, no study of this kind has ever been conducted in Taiwan. Although we previously reported that breast milk OCP levels in Taiwan are lower compared to those in other countries [1], low level exposure can still pose health risks, especially to newborns or infants. Thus, we aim herein to determine whether breastmilk OCP residues are associated with the neurodevelopment of infants as well as with cord blood thyroid and growth hormone levels among mother–breastfed infant pairs in Southern Taiwan. The best specimens for estimation of prenatal exposure to OCPs are maternal serum collected before delivery and umbilical cord blood [24,25]. Breast milk, which is a major food source for breastfeeding infants, is best used to represent postnatal exposure of infants to OCPs. Although OCP residues in breast milk were significantly and highly correlated with those in maternal serum and cord blood in several reports [24,25,26,27,28], OCP residues in breast milk do not totally reflect prenatal exposure of infants to OCPs. This study aims to determine the associations of breast milk OCP levels to cord blood thyroid and growth hormone levels as well as to the infant neurodevelopment.

## 2. Materials and Methods

The participants in the present study were healthy mother–infant pairs recruited from local hospitals in southern Taiwan from April 2007 to April 2010. The protocol of this study was evaluated and approved by the institutional review boards of the Human Ethical Committees of Pingtung Christian Hospital (PCH) in 2007 (NO: IRB021). The participants in this study were selected and recruited as described in detail in our previous studies [29,30]. Our subjects were pregnant women in southern Taiwan without any clinical complications recruited between April 2007 and July 2010. At the onset of the study, 358 pregnant women were randomly recruited. Only 143 subjects offered sufficient milk and completely answered the detailed questionnaire. Our research assistants contacted all 143 subjects by telephone, and only 78 subjects agreed to participate in this study. The parameters of the birth outcomes were gathered, and the cord-blood levels of the thyroid hormones (THs), including triiodothyronine (T3), thyroxine (T4), TSH, free T3 (FT3), free T4 (FT4), and growth hormone insulin-like growth factor 1 (IGF-1) were determined using an automated chemiluminescence analyzer (Architect i-2000) (Abbott Diagnostics, IL, USA) after delivery. We invited our subjects (mother–infant pairs) to be reviewed by pediatricians and to have their infants’ neurodevelopment at 8 to 12 months of age assessed by infant psychometrists. Seventy-eight participants agreed to join the program, and 55 mother–infant pairs were selected on the basis of exclusive or partial breastfeeding. The infants’ neurological and neurobehavioral development were tested using the Bayley-III scales, which consist of cognitive, language, motor, social–emotional, and adaptive behavior scales [31]. The Bayley-III composite scores are interpreted in a qualitative way to determine the child’s level of performance as assessed and characterized into different levels of performance: very superior (130 and above), superior (120–129), high average (110–119), average (90–109), low average (80–89), borderline (70–79), and extremely low (69 and below). Overall, scores that fall below 70 indicate poor performance. The Bayley-III is a tool used to measure the age-appropriate development of infants and children aged from one month to four years (the best period for assessment is from 6 months to 2.5 years), and it is designed to help parents and caregivers gain a better understanding of a child’s strengths and weaknesses (see the supplemental materials).

Several chemicals and reagents were used in this study as follows: The standards of the OCPs (EPA method 8081 organochlorine pesticide mixture) were purchased from AccuStandard Inc. (New Haven, CT, USA), while the internal standards of ^13^C_12_ 4,4′-DDT and pentachloro–nitrobenzene were obtained from Cambridge Isotope Laboratories (Tewksbury, MA, USA). Silica gel (100–200 mesh) was purchased from Merck (Darmstadt, Germany). All the solvents were pesticide residue grade from Tedia (Fairfield, OH, USA), Sigma-Aldrich (St. Louis, MO, USA), and Merck. The cartridges used included a 6 cc/5 mg Sep-Pak Vac C18 cartridge (Waters, Milford, MA, USA), a 360 mg Sep-Pak Plus NH_2_ cartridge (Waters) and a 3 cc/1 g Bond Elut PCB cartridge (Varian, Harbor City, CA, USA) for solid phase extraction. For the sample collection, milk samples were collected from the subjects within two weeks or the first month after giving birth. The samples were gathered in chemical-free glass bottles and stored frozen in the subjects’ home refrigerators. For each sample, a volume of more than 60 mL was collected and immediately transferred to the laboratory at National Pingtung University of Science and Technology. The samples were kept frozen at −20 °C prior to OCP analysis. The OCP residues in the breast milk were collected and extracted. Afterwards, the samples were subjected to clean-up and analysis as previously described [1,11]. Twenty different OCPs, including 4,4′-dichlorodiphenyldichloroethane (DDD), 4,4′-DDE, 4,4′-DDT, α-HCH, β-HCH, γ-HCH, δ-HCH, cis-CHL, trans-CHL, heptachlor, heptachlor epoxide, aldrin, endrin, endrin aldehyde, endrin ketone, dieldrin, decachlorobiphenyl, endosulfan I (a), endosulfan II (b), endosulfan sulfate, and methoxychlor, were determined using high resolution gas chromatography/mass spectrometry in the splitless mode (Agilent 7890/5975C-GC/MSD from Hewlett-Packard, Palo Alto, CA) and a capillary column (DB-5MS purchased from J&W Scientific, Folsom, CA) separated in the electron impact (EI) mode. The sample quality assurance and quality control (QA/QC) followed the Taiwan Environmental Protection Agency (EPA) standard method (NIEA T206.21). For the sample quality assurance and quality control (QA/QC), we followed the Taiwan Environmental Protection Agency (EPA) standard method (NIEA T206.21). A set of standard, blank, and pooled milk samples were inserted into each batch of approximately 10 samples to verify the accuracy and precision of each measurement. The recovery rates of two isotopically labeled standards (^13^C_12_-4,4′-DDT and pentachloronitrobenzene) ranged from 96.7% to 105%. The relative standard deviations of levels in the blind samples were less than 20% for all detectable compounds. The limits of detection (LODs) were defined as 3 times the signal-to-noise (S/N) ratio and with values between 0.0151 to 0.0540 ng/g lipid for the twenty OCPs.

For the cord blood THs and IGF-1 collection and analysis, cord blood samples from our subjects were collected by the obstetricians after delivery. Two tubes of blood with a volume of 10–15 mL each were drawn after delivery to be immediately examined for THs and IGF-1 levels using the chemiluminescence methods as described previously [30,32]. A blind duplicate was measured for every 10 samples. Levels of THs (T3, T4, TSH, FT3, and FT4) and IGF-1 in the umbilical cord blood samples were detected at the Clinical Laboratory of PCH using chemiluminescence immunoassay kits purchased from Diagnostic Products Corporation (Los Angeles, CA, USA) and were measured using an automated chemiluminescence analyzer (Architect i-2000) (Abbott Diagnostics, Abbott Park, IL, USA) as per the instructions issued by the manufacturing company.

The data for the OCPs lower than the method of detection limits (MDLs) were set to half the MDL for further statistical analysis. Nonparametric methods, Spearman’s correlation coefficients, and Mann–Whitney U tests were used to examine the between-group differences and relationships. The individual OCP compound was dichotomized at the 75th percentile into higher (>75th percentile) and lower (≤75th percentile) exposure groups to do further statistical analyses. The Bayley-III scales were converted into two groups based on whether the scores were over or below average (90–109) except for the cognitive scale. The cognitive scale used the median as the cutoff point due to only having four cases with below average composite scores. The odds ratios (ORs) of Bayley-III scales composite scores were examined using logistic regression models with/without adjustment of maternal age, pre-pregnant BMI, parity, and infant gender. Multivariate analyses including a multivariate analysis of covariance (MANCOVA) and a redundancy analysis (RDA) were used to determine associations between the Bayley-III scores and individual OCP or THs and IGF-1. Several studies have reported that serum or breastmilk levels of OCPs are significantly and positively correlated with women’s age; inversely, OCP levels have been significantly and negatively linked to women’s BMI and parity [1,11,33,34]. The variables, including maternal age, pre-pregnant BMI, parity, and infant gender, were considered as the covariates to do the further statistical analyses, especially for the purpose of adjustment. All statistical analyses were performed using Statistical Product and Service Solutions, version 12.0, except for RDA. RDA was examined by Microsoft Excel^®^ XLSTAT^TM^ (Addinsoft, rue Damrémont, Paris, France).

## 3. Results

Table 1 shows the descriptive statistics of the study participant-pairs (*n* = 55) in the present study along with the demographic parameters of the mothers and newborns, the various thyroid and growth hormone levels determined from the cord blood, and the infants’ developmental assessment scores according to the Bayley-III. More descriptive information can be found in our supplemental material (see Tables S1–S3). The breast milk levels of 20 individual OCPs in ng/g lipid are shown in Table 2 with 4,4′-DDE having the highest mean ± SD of 10.3 ± 6.76, followed by 4,4′-DDD (1.00 ± 1.43), and 4,4′-DDT (0.715 ± 0.745). Similar to our previous studies [1,11], 4,4′-DDE was found to be the predominant OCP residue in the breast milk. DDE is the major metabolite of DDT and has a longer half-life in the human body compared to DDT and its other degradation products. After the DDTs, the only breast milk OCPs with levels detected above 0.3 ng/g lipid were heptachlor (0.660 ± 0.685 ng/g lipid), heptachlor epoxide (0.365 ± 0.388 ng/g lipid), aldrin (0.366 ± 0.474 ng/g lipid), dieldrin (0.352 ± 0.422 ng/g lipid), and endrin (0.313 ± 0.208 ng/g lipid). Of the 20 OCPs, only 4,4′-DDE and heptachlor had 100% detection rates in the 55 breast milk samples, while endosulfan II and methoxychlor had levels above the LODs in less than 50% of the samples.

Significant between-group differences were observed between δ-HCH levels in the breast milk and the dichotomized cognitive scale scores (*p* = 0.025) as shown in Table 3. Infants with lower cognitive scores (median ≤ 100) were significantly associated with lower levels of δ-HCH in the breast milk of their mothers. In terms of the language scale scores and the levels of breast milk 4,4′-DDD, 4,4′-DDT, dieldrin, endosulfan I, and endrin ketone, the higher (>89) and lower (≤89) groups also showed significant between-group differences (*p* < 0.05). Lower language scores were significantly associated with low levels of all of these OCPs except for breast milk 4,4′-DDT, which at low concentrations, is significantly linked to infants with higher language scores. Infants having social-emotional scores >89 were significantly associated with the breast milk of their mothers containing lower *trans*-CHL residue levels (*p* = 0.048). No significant between-group differences were observed for breast milk OCP levels and the dichotomized scores for the motor and adaptive behavior scales.

We compared cord blood hormone levels in infants with maternal OCP levels above and below the 75th percentile (Table 4). Higher breast milk α-HCH and endosulfan I residues were significantly associated with lower cord blood T3 levels (0.320 ± 0.0666 ng/mL, *p* = 0.010) as compared to lower breast milk concentrations of this residue. Likewise, higher breast milk α-HCH, β-HCH, and heptachlor residues were significantly associated with lower TSH levels in the cord blood. Similarly, higher α-HCH and heptachlor epoxide residues in the breast milk were significantly associated with lower levels of cord blood FT3. Higher breast milk β-HCH, *cis*-CHL, *trans*-CHL, 4,4′-DDD, and heptachlor epoxide residues were significantly associated with lower levels of the growth hormone IGF-1 in the cord blood. No significant differences were found between the high and low exposure breast milk OCP groups and cord blood levels of T4 and FT4. Overall, specific breast milk OCP exposure groups showed inverse relationship with the T3, TSH, Free T3, and IGF-1 levels in the cord blood. The correlations of the thyroid and growth hormone levels in the cord blood with the Bayley-III outcomes were also successively determined by performing the Spearman rho correlation coefficient test (Table S4). Cord blood T4 (r = 0.270, *p* = 0.041), FT4 (r = 0.271, *p* = 0.040), and IGF-1 (r = 0.383, *p* = 0.013) levels were significantly and positively correlated with better scores on the motor scale while cord blood T4 (r = 0.298, *p* = 0.027) and FT4 (r = 0.274, *p* = 0.038) levels were significantly and positively associated with a better adaptive behavioral performance, respectively.

Although 4,4′-DDT levels in the breast milk did not have any significant associations with cord blood thyroid and growth hormone levels, there were significant associations with the two Bayley outcomes based on the determined odds ratio values (ORs) in Table 5. This suggests a thyroid and growth hormone-independent mechanism related to how 4,4′-DDT residue levels in the breast milk might affect brain development in infants. Infants exposed to low levels of breast milk 4,4′-DDT were shown to have better cognitive (OR = 8.09, *p* = 0.025) and language (OR = 11.9, *p* = 0.013) performance than the infants with higher levels of exposure. Likewise, lower breast milk *trans*-CHL exposure in infants was associated with better performance in the social emotional area of the Bayley-III scale (OR = 6.06, *p* = 0.01).

**Figure 1:**
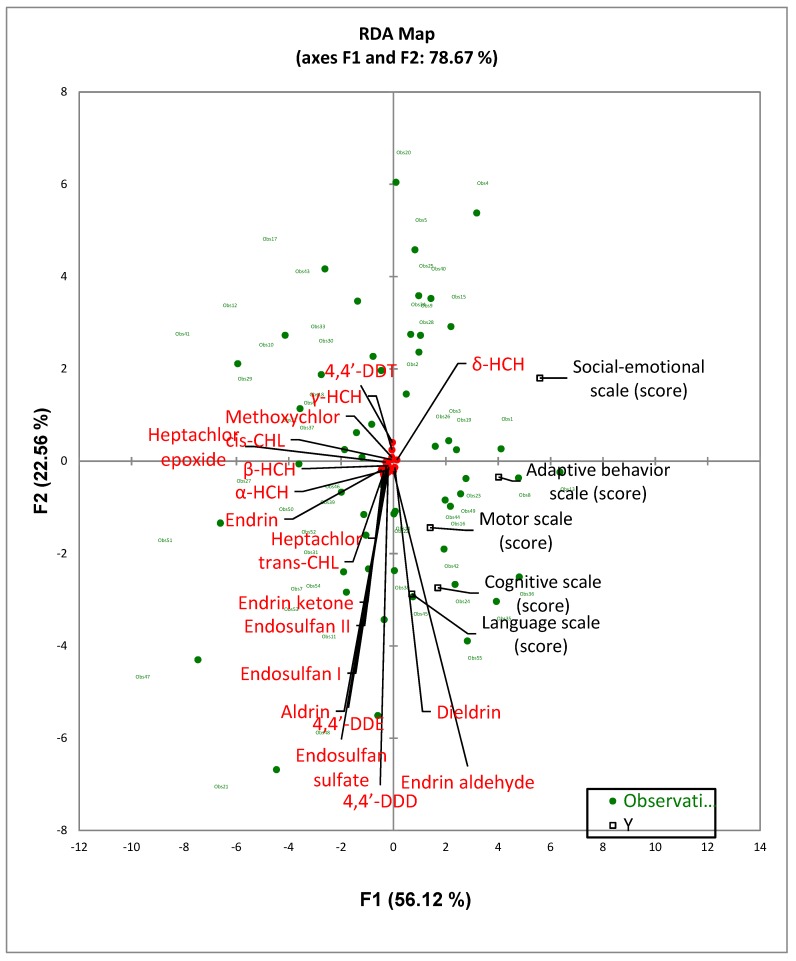
Redundancy analysis for the quantitative correlation results between 20 OCPs and the scores for the 5 Bayley-III subtests.

**Figure 2:**
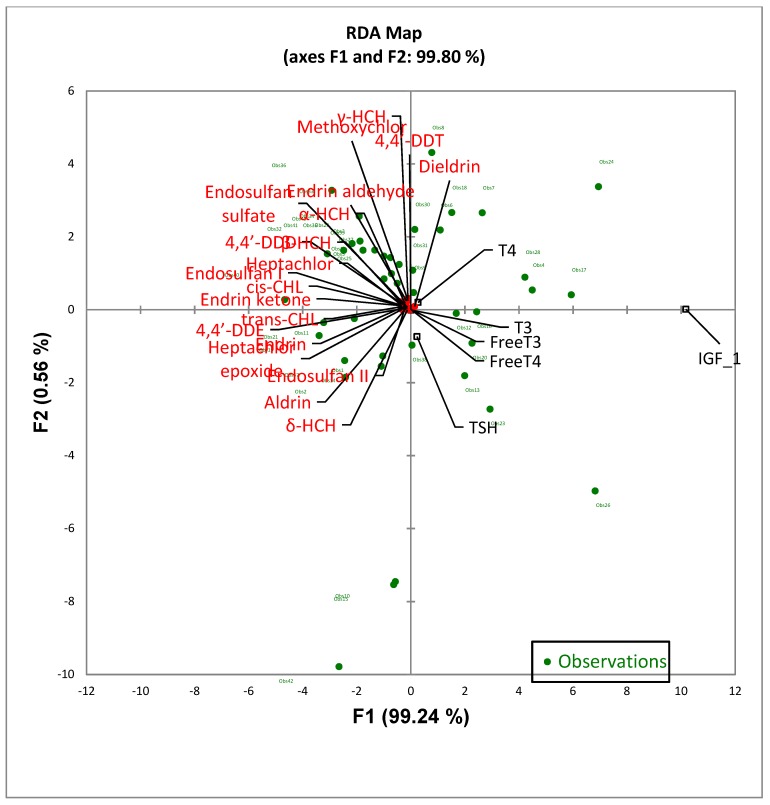
Redundancy analysis for the quantitative correlation results between 20 OCPs and the THs and IGF-1 expression.

For the multivariate analysis, the RDA and MANCOVA tests were used to determine and clearly visualize the relationship of the Bayley-III score outcomes and cord blood thyroid and growth hormones with the OCP levels in the breast milk samples as well as to predict the Bayley-III scores using the breast milk OCP levels (Figure 1, Figure 2 and Table 6). The RDA map, as shown in Figure 1, shows that the cognitive, language, and motor outcomes were significantly and highly correlated with breast milk OCPs. In addition, the social–emotional and adaptive behavior scores also showed a significant correlation with breast milk OCPs. All the breast milk OCPs except for dieldrin, δ-HCH, and endrin aldehyde, were negatively associated with the 5 Bayley-III domain scales. The RDA analysis results for endrin was found to be similar to that of the results from the Spearman rho correlation coefficient test in which it was found to have negative correlations with the social–emotional scale (r = −0.324, *p* = 0.016). Similar to the findings shown in Table 5, 4,4′-DDT was inversely linked to the cognitive and language scales, and trans-CHL had a negative correlation with the social–emotional scales in the RDA map. Figure 2 shows the RDA map for the associations between breast milk OCP levels and thyroid and growth hormone levels in the cord blood. More notable significant negative correlations between the growth hormone IGF-1 and most of the OCP residues detected in the breast milk samples were observed as compared to that of the thyroid hormones.

Certain OCPs were found to be able to predict the five domains of the Bayley-III scale using the MANCOVA test, as shown in Table 6. Breastmilk 4,4′-DDT, and endrin were negatively correlated with the cognitive scores after maternal age, pre-pregnant BMI, parity, and infant gender were adjusted. In the cognitive scale outcome, 4,4′-DDT significantly decreased the score to 7.43 (95% CI: −12.6–−2.29), and endrin decreased it to 14.2 (95% CI: −26.8–−1.56) with R^2^ = 0.328. Social–emotional outcomes had significantly inverse associations with Endrin (R^2^ = 0.16, β = −24.5, *p* = 0.041). Similarly, this association was also observed between the adaptive behavior outcome scale and heptachlor epoxide (R^2^ = 0.03, β = −31.4, *p* = 0.041), respectively. The motor scale (R^2^ = 0.255) was negatively and significantly linked to 4,4′-DDT (β = −6.13, *p* = 0.010) and heptachlor epoxide (β = −26.6, *p* = 0.001). However, OCPs such as heptachlor (β = 18.5, *p* = 0.010) and endosulfan I (β = 21.5, *p* = 0.015) were found to have significant positive associations with the motor and language scores. As compared to the RDA analysis, the MANCOVA test showed that higher heptachlor exposure was associated with better motor performance whereas higher endosulfan I exposure was correlated with better language score. All other breast milk OCPs showed consistent significant negative associations with the Bayley outcomes except for heptachlor and endosulfan I for the RDA and MANCOVA analyses.

## 4. Discussion

In infant neurodevelopment, according to Montgomery [35], newborns exhibit a normal range of Apgar scores ranging from 7 to 10. Apgar scores obtained 1- and 5-min following birth were within the normal range in the current investigation. However, Apgar scores cannot totally predict neurological and neurobehavioral outcomes during early life, which is the reason why infants aged 8 to 12 months of age were assessed using the Bayley-III. With regards to the five developmental domains: cognitive, language, motor, social–emotional, and adaptive behavior [36], the average composite scores of the 55 assessed infants were within the normal range. Among the five domains of the Bayley-III, the three quantitative measurements, cognitive, language, and motor scales, as examined by the psychometrists in this study, were significantly correlated (r = 0.319–0.554, *p* < 0.01–0.018), as was the case for the other two scales based on the questionnaires answered by the parents providing the results for the social–emotional and adaptive behavior scales (r = 0.436, *p* = 0.001). Additionally, the scores from the two questionnaire-based scales were independent of those obtained from the other three scales, which were quantitative-based measurements in the present Bayley-III study (*p* > 0.05). However, there was an exception for the language and social–emotional scales, which showed a statistical correlation (r = 0.280, *p* = 0.038).

After adjustment for confounding factors such as maternal age, pre-pregnant BMI, parity, and infant gender, and using the logistic regression models in our study, we found that the breastmilk levels of 4,4′-DDT and trans-CHL were significantly and negatively correlated with the cognitive, language, and social-emotional scores. Higher breast milk levels of 4,4′-DDT or *trans*-CHL were found to be significantly associated with poor performance in the cognitive, language, and social–emotional domains in this study. In addition, the RDA analysis showed that our selected breastmilk OCPs, including 4,4′-DDT, endrin, endosulfan I, heptachlor, and heptachlor epoxide, were inversely related with all five domains of the Bayley-III scales. Similarly, the MANCOVA test also showed negative correlations between the selected breast milk OCPs and the Bayley outcomes, except for endosulfan I and heptachlor, which showed significant positive correlations with the language and motor scale. DDT and cis-CHL have already been banned for use for more than three decades in Taiwan. However, adverse neurological outcomes for infants were observed based on our findings. Although 4,4′-DDE levels appeared to be higher as compared to other OCPs, no correlations were found between 4,4′-DDE concentrations in the breast milk and the Bayley outcomes in this study. DDT and its metabolites, such as DDE and DDD, have been reported to cross the placenta and contaminate breast milk, resulting in neurodevelopmental-related toxicities [4,10,37,38]. Eskenazi et al. [39] investigated prenatal exposure to maternal serum DDT and DDE in a malaria control area and reported that diminution of mental development in infants aged 12 to 24 months and impairment of psychomotor development in infants aged 6 to 12 months were associated with the presence of DDT and DDE. Four-year-old participants in a study conducted in Spain were observed to have cord blood serum DDT levels associated with their quantitative and memory and perceptual performance skill scores [40]. In a national birth cohort study performed from 1987 to 2005, the Finnish Prenatal Study of Autism (FiPS-A), maternal serum levels of p,p′-DDE were found to be associated with autism spectrum disorder (ASD) in 75 offspring [41,42]. The presence of high chlordecone levels in breast milk and cord blood samples in 18-month-old infants was observed to be associated with neuronal impairments such as poorer fine motor scores among boys [16]. Additionally, another study conducted by Roberts et al. [43] reported an association with ASD in first trimester mothers exposed to OCPs such as DDT and Dicofol. As for endosulfan I and heptachlor, no study yet has reported on their associations with infant neurodevelopmental outcomes. The inconsistency in the results of the RDA and MANCOVA tests, particularly in terms of the correlations between breast milk OCPs such as endosulfan I and heptachlor and the Bayley-III outcomes, may be attributed to several covariates or confounding factors that were not taken into consideration in the present study. The first candidates of covariates are OCPs or other chemicals like PBDEs in cord blood. In our previous study, breastmilk PBDEs were associated with disruption of thyroid hormones, IGF-1, and infant neurodevelopment [29,30]. The second candidates of covariates are thyroid and growth hormones or other hormones like estrogen or androgen in cord blood. OCPs as a class of endocrine-disrupting chemicals (EDCs) were found to induce low estrogenic activity. Also, the breastfeeding quantity and duration might have been covariates in relation to infant neurodevelopment when the breastfed infants were postnatally exposed to OCPs.

In our study, cord blood thyroid hormones and IGF-1 in mothers with higher OCP levels in the breast milk were lower in magnitude as compared with those in lactating mothers with lower levels of OCPs. Although breast milk OCPs do not represent prenatal exposure, they are highly and notably related with OCPs in maternal and cord blood. Specific OCPs have been reported to be associated with hormonal changes in sensitive mothers and offspring [44,45]. We observed that 4,4′-DDT and 4,4′-DDE levels in our breast milk samples did not show any significant associations with the thyroid and growth hormone levels in the cord blood samples. Similarly, Chevrier et al. [46] observed that p,p′-DDT, o,p′-DDT, and p,p′-DDE levels in the serums samples collected from women who enrolled in the birth cohort study between the period of October 1999 and October 2000 did not show any significant associations with thyroid hormone levels. However, other studies observed significant associations, including the observation that maternal total T3 levels were inversely linked to OCPs including p,p′-DDE, *cis*-nanochlor, and HCB detected in blood samples collected from women in 2005 [47]. In addition, Freire et al. [48] also found p,p′-DDE exposure to be associated with marginally-significantly higher odds of TSH ≥ 5 mU/L (OR = 1.32; 95% CI = 0.95, 1.83; *p* = 0.09), while HCB levels (β = −0.15; 95% CI = −0.31, 0.02; *p* = 0.09) were inversely associated with TSH. Newborn cord blood samples from Chinese infants were reported to have inversely associated FT4 levels although the findings were only marginally-significant in the case of ƩHCH, p,p′-DDE, and methoxychlor concentrations [23]. Maervoet et al. [44] reported that FT3 and FT4 are inversely associated with organochlorine compounds detected in cord blood samples collected from neonates [44]. Higher congenital hypothyroidism has been associated with elevated CHL levels detected in breastmilk samples [49]. β-HCH was reported to be significantly correlated with elevated levels of TSH in maternal cord serum [50]. Higher ORs of cord blood TSH levels ≥5 mU/L (80th percentile) (OR = 2.05; 95% CI = 1.01, 4.18; *p* = 0.05) were observed in newborn Spanish boys with high exposure to endrin, while lower ORs of TSH ≥ 5 mU/L (OR = 0.36; 95% confidence interval (CI) = 0.17, 0.77; *p* = 0.008) have been found to be negatively associated with higher endosulfan–sulfate exposure during the prenatal stage [48]. High levels of ΣHCHs (α, β, and γ isomers) have been found in females with euthyroidism and hypothyroidism [45]. Goldner et al. [51] reported CHL (OR = 1.3; 95% CI = 0.99, 1.7) and heptachlor (OR = 1.2; 95% CI = 0.66, 2.3) to be associated with hypothyroid disease in the female spouses of pesticide applicators from Iowa and North Carolina who participated in the 1993–1997 Agricultural Health Study after adjustment for their thyroid status, pesticide use, smoking status, hormone replacement therapy history, and age. Studies on the effects of OCP exposure to growth factors in cord blood serum samples have been limited. Boada et al. [52] reported a significant inverse relationship of IGF-1 and aldrin in serum samples from female women of Spanish decent. Overall, most scientific reports on environmental epidemiological topics, including ours, have found negative associations between thyroid hormones and IGF-1 with OCP exposure. Although the 4,4′-DDT exposure groups showed no significant associations with the cord blood thyroid and growth hormone levels (Table 4), its significant associations with the Bayley outcomes might be indicative of a thyroid hormone-independent mechanism related to developmental effects on the brain.

THs, such as T3/free T3, T4/free T4, and TSH, are critical, not only for day-to-day metabolism and homeostasis but also for the growth and development of mammals, including fetuses and infants, especially in the case of neurodevelopment [53]. The maintenance of the TH levels within a narrow range, especially in the pre- and early postnatal period, is critical for normal brain development [54]. Previous studies have demonstrated the importance of thyroid and growth hormones in the neurodevelopment of infants (see supplemental material) [54,55]. In our study, cord blood T4, Free T4, or TSH was positively linked to either motor or adaptive behavior outcomes. Only a few studies have reported the relevance of OCP exposure to thyroid hormones in infant neurodevelopment. Li et al. [56] investigated the maternal serum association between prenatal exposure to OCPs and the mental and psychomotor development of infants at ages 6 and 18 months. They indicated that hexachlorobenzene (HCB) was negatively associated with TSH in cord serum. For heptachlor epoxide, Yamazaki et al. [57] also found a significant inverse association between prenatal low-level exposure to *cis*-heptachlor epoxide and the MDI at the age of 18 months, as evaluated using the BSID-II in Japan [57]. In the case of the growth hormone IGF-1, few reports have examined the correlations between OCPs in cord blood or breast milk and cord blood IGF-1 in neonates, particularly for newborns. Zumbado et al. [58] revealed significant associations between 4,4-DDE and 4,4′-DDT and serum IGF-1 in male adolescents and boys. The findings of the present study pointed to the possibility that certain OCPs have potential effects on negative modulation of the TH/IGF-1 systems if the breastmilk OCPs in this study could partially reflect their levels in cord blood or maternal serum during pregnancy. The negative modification of TH/IGF-1 systems in fetuses may be linked to the properties of EDCs, under which OCPs are classified. T4, free T4, and IGF-1 in cord blood were positively correlated with the Bayley-III outcomes, while cord blood thyroid hormones and IGF-1 were found to positively affect infant development, including neurodevelopment, and there was an inverse correlation between breastmilk OCPs and the Bayley-III scores. This suggests that the activities of EDCs like OCPs in breast milk or cord blood, including their negative effects on the regulation of estrogen and androgens, could lead to significant disruptions in neurodevelopmental outcomes in 1-year-old infants. Consequently, hormonal disruption may also affect other behaviorally-related pathways such as the dopamine system. Dopamine is partially responsible for moods and behavior, including skills related to decision making and reinforced learning [59], which are both very important aspects of an individual’s capacity to adapt. In our study, heptachlor epoxide showed the highest inverse association with the Bayley-III adaptive behavior scale. Animal studies have shown that exposure to heptachlor is linked to disruptions in the dopamine system [54,60]. It only takes a matter of hours for approximately 20% of the heptachlor in the body to be degraded to heptachlor epoxide [60]. In other studies, heptachlor exposure has been associated with neurodegenerative and neurobehavioral disorders such as Parkinson’s disease [61] and depression [62], respectively. To this end, the presence of specific OCP levels in breast milk has been observed to have associations with the thyroid hormone levels in cord blood samples, which may be suggestive of maternal prenatal levels and fetal exposure. Several reports have indicated that infants with prenatal exposure to OCPs may experience disruption of thyroid hormones or neurological development. Neonatal TSH levels have been observed to be associated in newborns with neurodevelopmental impacts in relation to prenatal exposure to 17 OCPs [48]. The resin triiodothyronine uptake ratio (T3RU), which is a sensitive marker for the assessment of the thyroid function was found to be inversely associated with exposure to OCPs, such as PCBs, HCB, p,p′-DDE, and *trans*-nonachlor, during pregnancy and following birth and was subsequently reported to be positively associated with neurotoxic outcomes in preschool children [63]. In the present study, although 4,4′-DDE had the highest concentration among the detected breastmilk OCPs, no significant between-group differences in levels of thyroid and growth hormones were observed in the high and low DDE exposure groups, nor was it significantly associated with the Bayley outcomes. Overall, it was observed that T4, free T4, and IGF-1 in cord blood have significant positive associations with Bayley-III domains such as the motor and adaptive behavior scales. The results of the current study did not prove whether in-utero exposure to OCPs affected cord blood thyroid and growth hormones even though OCPs were reported in previous published articles to be highly correlated among maternal blood, cord blood, and breast milk samples [24,25,26,27,28].

There are some limitations in the present study that should be acknowledged aside from the fact that our sampling size was small (*n* = 55). One example is that our study did not consider the other confounding factors like the lactational exposure metric, which is included in breastfeeding quantity and duration. In addition, we didn’t consider if the mothers exclusively or partially breastfed their nursing infants. Although cord blood OCPs were not detected in the present study, breastmilk OCP levels could partially reflect or show the associations between prenatal and postnatal exposure to organochlorines and infant neurodevelopment. Nevertheless, we observed significant associations between OCPs and thyroid hormone, IGF-1, and neurodevelopment, even at low levels of exposure.

Finally, the breastmilk levels of OCPs in Taiwan were within the safe criteria based on the recommendations of the World Health Organization (WHO) and Environmental Protection Agency (EPA) and also as compared to other countries’ levels (Table S5). Significant inverse associations between breastmilk indicating low exposure levels to OCP and thyroid hormones, IGF-1, and infant neurodevelopment were found in the current study. Although OCPs have been banned for use in most developed countries for more than three decades, associated adverse effects may still be found in early life, including neonatal subjects and infants. There is still a need to conduct further studies to validate these associations and to determine the mechanism by which OCP exposure may lead to neurodevelopmental effects.

## 5. Conclusions

Breast milk OCPs such as 4,4′-DDT were found to have negative associations with Bayley cognitive and language outcomes, while *trans*-CHL was negatively associated with the social–emotional scale. RDA and MANCOVA analyses showed negative associations between most of the breast milk OCPs and the five domain Bayley-III scales. In addition, TSH and IGF-1 also showed inverse associations to certain breast milk OCPs. Although the 4,4′-DDE was the dominant OCP in the breast milk samples, no significant differences were observed between the high and low exposure groups and the thyroid and growth hormones in the cord blood. Also, 4,4′-DDT did not show any significant associations with the cord blood thyroid and growth hormones but it did show significant negative associations with the Bayley outcomes. Despite limitations of the study such as small sample size and lack of a direct measure of prenatal exposures, our studies suggest that early life exposure to OCPs negatively impacts neurodevelopment. Future studies are needed to identify critical windows of susceptibility to OCPs and their precise mechanism of action.

## Figures and Tables

**Table 1:** Descriptive statistics of the study participants-mother and infant pairs (*n* = 55).

Variables	Mean ± SD	Median	Range
Mothers			
Age (years)	29.5 ± 4.64	31.0	17.0–40.0
Prepregnant BMI (kg/m^2^)	22.5 ± 4.12	21.7	15.4–34.9
Parity (number)	1.91 ± 0.784	2.00	1.00–4.00
Length of residence in the Kaoping area (years)	22.2 ± 11.6	27.0	1.00–40.0
Newborns			
Demography			
Gestational age (week)	38.2 ± 1.47	38.0	32.0–40.0
Weight (kg)	3.09 ± 0.379	3.01	2.30–4.00
Length (cm)	49.1 ± 1.63	49.0	46.0–52.0
Head circumference (cm)	33.5 ± 1.19	33.0	30.5–36.0
Chest circumference (cm)	32.3 ± 1.41	32.0	29.5–35.0
Hormones in cord blood			
Cord blood T3 (ng/mL)	0.385 ± 0.0973	0.390	0.250–0.600
Cord blood T4 (μg/dL)	8.74 ± 1.56	8.83	5.22–12.5
Cord blood TSH (μIUg/mL)	4.85 ± 2.34	4.14	1.83–11.6
Cord blood FT3 (pg/mL)	1.38 ± 0.191	1.40	1.00–1.69
Cord blood FT4 (ng/dL)	1.11 ± 0.120	1.13	0.840–1.48
Cord blood IGF-1 (ng/mL)	58.8 ± 28.1	52.3	11.4–118
Neurodevelopment			
Newborns			
Apgar score at 1 min (score)	8.66 ± 0.601	9.00	6.00–10.0
Apgar score at 5 min (score)	9.02 ± 0.212	9.00	7.00–10.0
Infants (Bayley-III)			
Cognitive scale (score)	101 ± 10.3	100	85.0–125
Language scale (score)	100 ± 10.2	100	77.0–125
Motor scale (score)	97.9 ± 8.65	97.0	79.0–121
Social-emotional scale (score)	98.5 ± 18.2	100	55.0–140
Adaptive behavior scale (score)	98.6 ± 14.3	100	62.0–131
Age at the time of testing (month)	11.1 ± 1.01	11.0	8.00–12.0

**Table 2:** Residue levels of OCPs in breast milk from subjects in southern Taiwan (ng/g lipid) (*n* = 55).

OCPs	Mean ± SD ^a^	Median	Range	*N* > LODs ^b^
HCHs ^c^				
α-HCH	0.236 ± 0.310	0.126	<LOD–1.64	50.0
β-HCH	0.208 ± 0.241	0.117	<LOD–1.31	50.0
γ-HCH	0.128 ± 0.113	0.0922	0.600–0.110	48.0
δ-HCH	0.148 ± 0.122	0.108	<LOD–0.470	45.0
CHLs ^d^				
*cis*-CHL	0.113 ± 0.129	0.0765	<LOD–0.730	43.0
*trans*-CHL	0.131 ± 0.268	0.0669	<LOD–1.90	28.0
DDTs ^e^				
4,4′-DDD	1.00 ± 1.43	0.211	<LOD–5.56	31.0
4,4′-DDE	10.3 ± 6.76	9.84	0.350–44.6	55.0
4,4′-DDT	0.715 ± 0.745	0.391	<LOD–2.87	47.0
ΣEndosulfan ^f^				
Endosulfan I	0.194 ± 0.353	0.0905	<LOD–2.20	36.0
Endosulfan II	0.151 ± 0.367	0.0161	<LOD–2.51	25.0
Endosulfan sulfate	0.147 ± 0.179	0.0812	<LOD–0.740	47.0
ΣEndrin ^g^				
Endrin	0.313 ± 0.208	0.168	<LOD–3.04	51.0
Endrin ketone	0.112 ± 0.146	0.0194	<LOD–0.670	29.0
Endrin aldehyde	0.152 ± 0.265	0.0564	<LOD–1.56	38.0
ΣHeptachlor ^h^				
Heptachlor	0.660 ± 0.685	0.385	0.040–3.48	55.0
Heptachlor epoxide	0.365 ± 0.388	0.226	<LOD–1.53	52.0
Aldrin	0.366 ± 0.474	0.169	0.0100–2.32	51.0
Dieldrin	0.352 ± 0.422	0.172	0.0100–1.90	53.0
Methoxychlor	0.106 ± 0.149	0.0123	<LOD–0.620	26.0

^a^ Means standard deviations. ^b^ *N* > LODs: numbers higher than limits of detection (LODs). ^c^ HCHs: isomers of hexachlorocyclohexane. ^d^ CHLs: isomers of chlordane. ^e^ DDTs: 4,4′-dichlorodiphenyltrichloroethane (4,4′-DDT) and its metabolites, 4,4′-dichloro-diphenyl-dichloroethane (DDD) and 4,4′-dichlorodiphenyldichloro-ethylene (4,4′-DDE). ^f^ ΣEndosulfan: the sum of Endosulfan I, Endosulfan II, and Endosulfan sulfate. ^g^ ΣEndrin: Endrin and its derivatives, Endrin ketone and Endrin aldehyde. ^h^ ΣHeptachlor: Heptachlor and its metabolite (Heptachlor epoxide).

**Table 3:** Between-group differences in three domain scores of binary Bayley-III for specific OCPs in breast milk ^a^.

OCPs	Cognitive Scale			Language Scale			Social-Emotional Scale		
	≤median (*n* = 35)	>median (*n* = 20)	*p*	≤89 (*n* = 8)	>89 (*n* = 47)	*p*	≤89 (*n* = 17)	>89 (*n* = 38)	*p*
δ-HCH	0.124 ± 0.115	0.189 ± 0.125	0.025 *	0.213 ± 0.181	0.137 ± 0.107	0.417	0.129 ± 0.115	0.157 ± 0.125	0.465
trans-CHL	0.108 ± 0.108	0.173 ± 0.425	0.280	0.0456 ± 0.0353	0.146 ± 0.287	0.294	0.241 ± 0.445	0.0824 ± 0.105	0.048 *
4,4′-DDD	0.759 ± 1.07	1.43 ± 1.86	0.176	0.386 ± 1.05	1.11 ± 1.46	0.031 *	1.37 ± 1.56	1.35 ± 0.219	0.250
4,4′-DDT	0.856 ± 0.839	0.502 ± 0.495	0.298	1.49 ± 1.13	0.582 ± 0.578	0.015 *	0.733 ± 0.836	0.706 ± 0.713	0.898
Dieldrin	0.314 ± 0.362	0.418 ± 0.514	0.582	0.114 ± 0.0814	0.392 ± 0.444	0.042 *	0.274 ± 0.274	0.386 ± 0.473	0.792
Endosulfan I	0.129 ± 0.183	0.308 ± 0.522	0.411	0.0425 ± 0.0346	0.220 ± 0.376	0.019 *	0.222 ± 0.515	0.182 ± 0.258	0.852
Endrin ketone	0.0986 ± 0.128	0.133 ± 0.174	0.394	0.0298 ± 0.0295	0.125 ± 0.154	0.036 *	0.120 ± 0.188	0.107 ± 0.126	0.466

* *p* < 0.05. ^a^ Higher and lower groups were based on a cutoff point of 89, which is a critical value between average and lower than average Bayley-III scores, with the exception of the cognitive scale. The cutoff point for the cognitive scale is a median of 100.

**Table 4:** Between-group differences in cord blood thyroid (T3, T4, TSH, FT3, and FT4) and growth (IGF-1) hormone levels for specific OCPs in breast milk ^a^.

OCPs	Levels (ng/g Lipid)	T3 (ng/mL)	T4 (µg/dL)	TSH (µgU/mL)	FT3 (pg/mL)	FT4 (ng/dL)	IGF-1 (ng/mL)
α-HCH	≤0.269 (*n* = 40)	0.407 ± 0.0962	9.05 ± 1.67	5.34 ± 2.35	1.43 ± 0.185	1.12 ± 0.126	60.9 ± 26.6
	>0.269 (*n* = 15)	0.320 ± 0.0666	8.15 ± 1.31	3.78 ± 2.03	1.28 ± 0.175	1.09 ± 0.111	53.4 ± 32.3
	*p* value	0.010 *	0.153	0.004 **	0.018 *	0.612	0.328
β-HCH	≤0.260 (*n* = 40)	0.400 ± 0.0959	8.93 ± 1.69	5.03 ± 2.33	1.39 ± 0.207	1.11 ± 0.126	63.7 ± 29.0
	>0.260 (*n* = 15)	0.339 ± 0.0876	8.58 ± 1.18	4.52 ± 2.50	1.40 ± 0.149	1.12 ± 0.112	42.6 ± 17.7
	*p* value	0.084 ^#^	0.474	0.008 **	0.368	0.524	0.011 *
*cis*-CHL	≤0.142 (*n* = 40)	0.381 ± 0.102	8.87 ± 1.73	5.10 ± 2.46	1.38 ± 0.200	1.12 ± 0.129	65.5 ± 30.5
	>0.142 (*n* = 15)	0.391 ± 0.0818	8.72 ± 1.04	4.51 ± 2.12	1.40 ± 184	1.10 ± 0.108	44.9 ± 15.2
	*p* value	0.572	0.481	0.368	0.923	0.751	0.019 *
*trans*-CHL	≤0.125 (*n* = 40)	0.380 ± 0.0952	8.92 ± 1.40	5.08 ± 2.31	1.42 ± 0.173	1.13 ± 0.115	63.4 ± 25.9
	>0.125 (*n* = 15)	0.369 ± 0.104	8.45 ± 2.17	4.41 ± 2.50	1.29 ± 0.226	1.05 ± 0.129	45.4 ± 31.1
	*p* value	0.504	0.406	0.169	0.069 ^#^	0.078 ^#^	0.016 *
4,4′-DDD	≤1.88 (*n* = 40)	0.398 ± 0.103	9.01 ± 1.61	5.10 ± 2.34	1.42 ± 0.187	1.12 ± 0.123	64.7 ± 29.2
	>0.1.88 (*n* = 15)	0.346 ± 0.0675	8.27 ± 1.56	4.42 ± 2.40	1.30 ± 0.191	1.10 ± 0.122	43.6 ± 18.5
	*p* value	0.169	0.214	0.145	0.071 ^#^	0.825	0.035 *
Endosulfan I	≤0.190 (*n* = 40)	0.402 ± 0.0901	8.77 ± 1.54	5.19 ± 2.48	1.41 ± 0.195	1.11 ± 0.105	60.9 ± 26.3
	>0.190 (*n* = 15)	0.329 ± 0.0992	8.91 ± 1.89	4.06 ± 1.73	1.33 ± 0.188	1.11 ± 0.168	51.8 ± 34.1
	*p* value	0.015 *	0.789	0.153	0.259	0.708	0.237
Endrin	≤0.375 (*n* = 40)	0.388 ± 0.0985	8.98 ± 1.54	5.21 ± 2.41	1.40 ± 0.200	1.13 ± 0.120	59.7 ± 23.7
	>0.375 (*n* = 15)	0.370 ± 0.0945	8.34 ± 1.76	4.11 ± 2.06	1.34 ± 0.177	1.05 ± 0.111	56.5 ± 38.4
	*p* value	0.630	0.161	0.075	0.354	0.059 ^#^	0.384
Heptachlor	≤1.04 (*n* = 40)	0.401 ± 0.101	9.00 ± 1.66	5.34 ± 2.53	1.40 ± 0.212	1.12 ± 0.127	63.4 ± 28.4
	>1.04 (*n* = 15)	0.336 ± 0.0678	8.29 ± 1.41	3.78 ± 1.28	1.36 ± 0.136	1.08 ± 0.103	47.0 ± 24.7
	*p* value	0.059 ^#^	0.186	0.033 *	0.382	0.255	0.071 ^#^
Heptachlor epoxide	≤0.501 (*n* = 40)	0.395 ± 0.101	9.11 ± 1.55	5.20 ± 2.29	1.42 ± 0.189	1.13 ± 0.115	64.8 ± 27.2
	>0.501 (*n* = 15)	0.355 ± 0.0825	8.08 ± 1.59	4.24 ± 2.44	1.30 ± 0.182	1.07 ± 0.131	45.0 ± 25.9
	*p* value	0.308	0.105	0.052 ^#^	0.046 *	0.189	0.012 *

^#^*p* < 0.1, * *p* < 0.05, ** *p* < 0.01. ^a^ Higher and lower groups were based on a cutoff point of the 75th percentile for OCPs.

**Table 5:** Odds ratios of binary Bayley scores between lower exposure (OCP levels ≤75th percentile) and higher exposure (OCP levels >75th percentile) groups.

**OCPs** **(ng/g Lipid)**	**Cognitive Scales (Score)**					
	**≤median ^a^**	**>median**	**Odds Ratios**	**95% C.I. ^b^**	**Adjusted ^c^**	**Crude**
	**(*n* = 35)**	**(*n* = 20)**	**(ORs)**		***p* Value**	***p* Value**
4,4’-DDT					0.025 *	0.061
>1.04 (*n* = 14)	12/14	2/14	1.00	1.00		
≤1.04 (*n* = 41)	23/41	18/41	8.09	1.30–50.3		
**OCPs** **(ng/g Lipid)**	**Language Scales (Score)**					
	**≤89 ^d^**	**>89**	**Odds Ratios**	**95% C.I. ^b^**	**Adjusted ^c^**	**Crude**
	**(*n* = 8)**	**(*n* = 47)**	**(ORs)**		***p* Value**	***p* Value**
4,4’-DDT					0.013 *	0.017 *
>1.04 (*n* = 14)	5/14	9/14	1.00	1.00		
≤1.04 (*n* = 41)	3/41	38/41	11.9	1.69–84.6		
**OCPs** **(ng/g Lipid)**	**Social Emotional Scales (Score)**					
	**≤89 ^d^**	**>89**	**Odds Ratios**	**95% C.I. ^b^**	**Adjusted ^c^**	**Crude**
	**(*n* = 17)**	**(*n* = 38)**	**(ORs)**		***p* Value**	***p* Value**
trans-CHL					0.010 *	0.010 *
>0.125 (*n* = 13)	8/13	5/13	1.00	1.00		
≤0.125 (*n* = 42)	9/42	33/42	6.06	1.55–23.8		

* *p* < 0.05. ^a^ The median was used as the cutoff point due to having only four cases with scores below 89. ^b^ 95% confidence interval. ^c^ Adjusted by maternal age, pre-pregnant BMI, parity, and infants’ gender. ^d^ meaning below average scores (90–109) including low average (80–89), borderline (70–79), and extremely low (69 and below).

**Table 6:** Bayley-III scores were predicted by OCP levels using multivariate linear regression models with adjustment for maternal age, pre-pregnant BMI, parity, and infant gender.

Developmental Domain Covariance	β-Estimated (95%CI)	*p* Value
Cognitive scale (score) ^a^		
4.4′-DDT	–7.43 (–12.6–−2.29)	0.007 **
Endrin	–14.2 (–26.8–−1.56)	0.029 *
Gender (female vs. male)	10.3 (3.53–17.1)	0.005 **
Language scale (score) ^b^		
Endosulfan I	21.5 (4.63–38.3)	0.015 *
Gender (female vs. male)	8.76 (2.67–14.8)	0.007 **
Motor scale (score) ^c^		
4.4′-DDT	–6.13 (−10.6–−1.65)	0.010 *
Heptachlor	18.5 (4.80–32.2)	0.010 *
Heptachlor epoxide	–26.6 (–41.7–−11.5)	0.001 **
Social-emotional scale (score) ^d^		
Endrin	–24.5 (–47.8–−1.12)	0.041 *
Adaptive behavior scale (score) ^e^		
Heptachlor epoxide	–31.4 (–61.4–−1.34)	0.041 *

* *p* < 0.05, ** *p* < 0.01; ^a^: adjusted R^2^ = 0.328, ^b^: adjusted R^2^ = 0.368, ^c^: adjusted R^2^ = 0.255, ^d^: adjusted R^2^ = 0.16, ^e^: adjusted R^2^ = 0.03; Generalized linear model adjusted for maternal age, parity, pre-pregnant BMI, gender.

## Supplementary Materials

The following are available online at https://www.mdpi.com/1660-4601/16/8/1438/s1, Table S1: The descriptive analysis in our participants (*n* = 55), Table S2: Spearman’s rho correlation coefficients (r) between neurodevelopmental outcomes and birth outcomes (*n* = 55), Table S3: Spearman’s rho correlation coefficients (r) between cord blood hormone levels and birth outcomes (*n* = 55), Table S4: Spearman’s rho correlation coefficients (r) between neurodevelopmental outcomes and cord-blood hormone levels (*n* = 55), Table S5: OCP levels in breast milk in different countries.

## References

[1] Chao H.R., Wang S.L., Lin T.C., Chung X.H. (2006). Levels of organochlorine pesticides in human milk from central Taiwan. Chemosphere.

[2] Tsai W.T. (2010). Current status and regulatory aspects of pesticides considered to be persistent organic pollutants (POPs) in Taiwan. Int. J. Environ. Res. Public Health.

[3] Ribas-Fito N., Cardo E., Sala M., Eulalia de Muga M., Mazon C., Verdu A., Kogevinas M., Grimalt J.O., Sunyer J. (2003). Breastfeeding, Exposure to Organochlorine Compounds, and Neurodevelopment in Infants. Pediatrics.

[4] ATSDR (Agency for Toxic Substances and Disease Registry) (2002). Toxicological Profile for DDT, DDE, and DDD.

[5] Longnecker M.P., Klebanoff M.A., Zhou H., Brock J.W. (2001). Association between maternal serum concentration of the DDT metabolite DDE and preterm and small-for-gestational-age babies at birth. Lancet.

[6] Aktar M.W., Sengupta D., Chowdhury A. (2009). Impact of pesticides use in agriculture: Their benefits and hazards. Interdiscip. Toxicol..

[7] Chang G.R. (2018). Persistent organochlorine pesticides in aquatic environments and fishes in Taiwan and their risk assessment. Environ. Sci. Pollut. Res. Int..

[8] Okoya A.A., Ogunfowokan A.O., Asubiojo O.I., Torto N. (2013). Organochlorine Pesticide Residues in Sediments and Waters from Cocoa Producing Areas of Ondo State, Southwestern Nigeria. ISRN Soil Sci..

[9] Mrema E.J., Rubino F.M., Brambilla G., Moretto A., Tsatsakis A.M., Colosio C. (2013). Persistent organochlorinated pesticides and mechanisms of their toxicity. Toxicology.

[10] Saeedi Saravi S.S., Dehpour A.R. (2016). Potential role of organochlorine pesticides in the pathogenesis of neurodevelopmental, neurodegenerative, and neurobehavioral disorders: A review. Life Sci..

[11] Chen M.-W., Santos H., Que D., Gou Y.-Y., Tayo L., Hsu Y.-C., Chen Y.-B., Chen F.-A., Chao H.-R., Huang K.-L. (2018). Association between Organochlorine Pesticide Levels in Breast Milk and Their Effects on Female Reproduction in a Taiwanese Population. Int. J. Environ. Res. Public Health.

[12] Gladen B.C., Ragan N.B., Rogan W.J. (2000). Pubertal growth and development and prenatal and lactational exposure to polychlorinated biphenyls and dichlorodiphenyl dichloroethene. J. Pediatr..

[13] Karmaus W., Asakevich S., Indurkhya A., Witten J., Kruse H. (2002). Childhood growth and exposure to dichlorodiphenyl dichloroethene and polychlorinated biphenyls. J. Pediatr..

[14] Gerhard I., Monga B., Krahe J., Runnebaum B. (1999). Chlorinated hydrocarbons in infertile women. Environ. Res..

[15] Muller M.H.B., Polder A., Brynildsrud O.B., Karimi M., Lie E., Manyilizu W.B., Mdegela R.H., Mokiti F., Murtadha M., Nonga H.E. (2017). Organochlorine pesticides (OCPs) and polychlorinated biphenyls (PCBs) in human breast milk and associated health risks to nursing infants in Northern Tanzania. Environ. Res..

[16] Boucher O., Simard M.N., Muckle G., Rouget F., Kadhel P., Bataille H., Chajes V., Dallaire R., Monfort C., Thome J.P. (2013). Exposure to an organochlorine pesticide (chlordecone) and development of 18-month-old infants. Neurotoxicology.

[17] Pan I.J., Daniels J.L., Goldman B.D., Herring A.H., Siega-Riz A.M., Rogan W.J. (2009). Lactational exposure to polychlorinated biphenyls, dichlorodiphenyltrichloroethane, and dichlorodiphenyldichloroethylene and infant neurodevelopment: An analysis of the pregnancy, infection, and nutrition babies study. Environ. Health Perspect..

[18] Meeker J.D., Altshul L., Hauser R. (2007). Serum PCBs, p,p′-DDE and HCB predict thyroid hormone levels in men. Environ. Res..

[19] Freire C., Koifman R.J., Sarcinelli P., Rosa A.C., Clapauch R., Koifman S. (2012). Long term exposure to organochlorine pesticides and thyroid function in children from Cidade dos Meninos, Rio de Janeiro, Brazil. Environ. Res..

[20] Alvarez-Pedrerol M., Ribas-Fito N., Torrent M., Carrizo D., Grimalt J.O., Sunyer J. (2008). Effects of PCBs, p,p′-DDT, p,p′-DDE, HCB and beta-HCH on thyroid function in preschool children. Occup. Environ. Med..

[21] Kim S., Park J., Kim H.J., Lee J.J., Choi G., Choi S., Kim S., Kim S.Y., Moon H.B., Kim S. (2013). Association between several persistent organic pollutants and thyroid hormone levels in serum among the pregnant women of Korea. Environ. Int..

[22] Dufour P., Pirard C., Seghaye M.C., Charlier C. (2018). Association between organohalogenated pollutants in cord blood and thyroid function in newborns and mothers from Belgian population. Environ. Pollut..

[23] Luo D., Pu Y., Tian H., Wu W., Sun X., Zhou T., Tao Y., Yuan J., Shen X., Feng Y. (2017). Association of in utero exposure to organochlorine pesticides with thyroid hormone levels in cord blood of newborns. Environ. Pollut..

[24] Darnerud P.O., Lignell S., Glynn A., Aune M., Tornkvist A., Stridsberg M. (2010). POP levels in breast milk and maternal serum and thyroid hormone levels in mother-child pairs from Uppsala, Sweden. Environ. Int..

[25] Jaraczewska K., Lulek J., Covaci A., Voorspoels S., Kaluba-Skotarczak A., Drews K., Schepens P. (2006). Distribution of polychlorinated biphenyls, organochlorine pesticides and polybrominated diphenyl ethers in human umbilical cord serum, maternal serum and milk from Wielkopolska region, Poland. Sci. Total Environ..

[26] Müller M.H.B., Polder A., Brynildsrud O.B., Grønnestad R., Karimi M., Lie E., Manyilizu W.B., Mdegela R.H., Mokiti F., Murtadha M. (2019). Prenatal exposure to persistent organic pollutants in Northern Tanzania and their distribution between breast milk, maternal blood, placenta and cord blood. Environ. Res..

[27] Tang-Peronard J.L., Heitmann B.L., Andersen H.R., Steuerwald U., Grandjean P., Weihe P., Jensen T.K. (2014). Association between prenatal polychlorinated biphenyl exposure and obesity development at ages 5 and 7 y: A prospective cohort study of 656 children from the Faroe Islands. Am. J. Clin. Nutr..

[28] Tsang H.L., Wu S., Leung C.K., Tao S., Wong M.H. (2011). Body burden of POPs of Hong Kong residents, based on human milk, maternal and cord serum. Environ. Int..

[29] Chao H.R., Tsou T.C., Huang H.L., Chang-Chien G.P. (2011). Levels of breast milk PBDEs from southern Taiwan and their potential impact on neurodevelopment. Pediatr. Res..

[30] Shy C.G., Huang H.L., Chao H.R., Chang-Chien G.P. (2012). Cord blood levels of thyroid hormones and IGF-1 weakly correlate with breast milk levels of PBDEs in Taiwan. Int. J. Hyg. Environ. Health.

[31] Lowe J.R., Erickson S.J., Schrader R., Duncan A.F. (2012). Comparison of the Bayley II Mental Developmental Index and the Bayley III Cognitive Scale: Are we measuring the same thing?. Acta Paediatr..

[32] Lin S.-M., Chen F.-A., Huang Y.-F., Hsing L.-L., Chen L.-L., Wu L.-S., Liu T.-S., Chang-Chien G.-P., Chen K.-C., Chao H.-R. (2011). Negative associations between PBDE levels and thyroid hormones in cord blood. Int. J. Hyg. Environ. Health.

[33] Brown A.S., Cheslack-Postava K., Rantakokko P., Kiviranta H., Hinkka-Yli-Salomäki S., McKeague I.W., Surcel H.-M., Sourander A. (2018). Association of Maternal Insecticide Levels With Autism in Offspring From a National Birth Cohort. Am. J. Psychiatry.

[34] Cohn B.A., Cirillo P.M., La Merrill M.A. (2019). Correlation of body mass index with serum DDTs predicts lower risk of breast cancer before the age of 50: Prospective evidence in the Child Health and Development Studies. J. Expo. Sci. Environ. Epidemiol..

[35] Montgomery K.S. (2000). Apgar Scores: Examining the Long-term Significance. J. Perinat. Educ..

[36] Weiss L.G., Oakland T., Aylward G.P. (2010). Bayley-III Clinical Use and Interpretation.

[37] Rogan W.J., Chen A. (2005). Health risks and benefits of bis(4-chlorophenyl)-1,1,1-trichloroethane (DDT). Lancet.

[38] Fenster L., Eskenazi B., Anderson M., Bradman A., Hubbard A., Barr D.B. (2007). In utero exposure to DDT and performance on the Brazelton neonatal behavioral assessment scale. Neurotoxicology.

[39] Eskenazi B., Marks A.R., Bradman A., Fenster L., Johnson C., Barr D.B., Jewell N.P. (2006). In Utero Exposure to Dichlorodiphenyltrichloroethane (DDT) and Dichlorodiphenyldichloroethylene (DDE) and Neurodevelopment Among Young Mexican American Children. Pediatrics.

[40] Ribas-Fitó N., Torrent M., Carrizo D., Muñoz-Ortiz L., Júlvez J., Grimalt J.O., Sunyer J. (2006). In utero exposure to background concentrations of DDT and cognitive functioning among preschoolers. Am. J. Epidemiol..

[41] Shen H., Main K.M., Kaleva M., Virtanen H., Haavisto A.M., Skakkebaek N.E., Toppari J., Schramm K.W. (2005). Prenatal organochlorine pesticides in placentas from Finland: Exposure of male infants born during 1997–2001. Placenta.

[42] Cheslack-Postava K., Rantakokko P.V., Hinkka-Yli-Salomaki S., Surcel H.M., McKeague I.W., Kiviranta H.A., Sourander A., Brown A.S. (2013). Maternal serum persistent organic pollutants in the Finnish Prenatal Study of Autism: A pilot study. Neurotoxicol. Teratol..

[43] Roberts E.M., English P.B., Grether J.K., Windham G.C., Somberg L., Wolff C. (2007). Maternal residence near agricultural pesticide applications and autism spectrum disorders among children in the California Central Valley. Environ. Health Perspect..

[44] Maervoet J., Vermeir G., Covaci A., Van Larebeke N., Koppen G., Schoeters G., Nelen V., Baeyens W., Schepens P., Viaene M.K. (2007). Association of thyroid hormone concentrations with levels of organochlorine compounds in cord blood of neonates. Environ. Health Perspect..

[45] Rathore M., Bhatnagar P., Mathur D., Saxena G.N. (2002). Burden of organochlorine pesticides in blood and its effect on thyroid hormones in women. Sci. Total Environ..

[46] Chevrier J., Eskenazi B., Holland N., Bradman A., Barr D.B. (2008). Effects of exposure to polychlorinated biphenyls and organochlorine pesticides on thyroid function during pregnancy. Am. J. Epidemiol..

[47] Takser L., Mergler D., Baldwin M., de Grosbois S., Smargiassi A., Lafond J. (2005). Thyroid Hormones in Pregnancy in Relation to Environmental Exposure to Organochlorine Compounds and Mercury. Environ. Health Perspect..

[48] Freire C., Lopez-Espinosa M.J., Fernandez M., Molina-Molina J.M., Prada R., Olea N. (2011). Prenatal exposure to organochlorine pesticides and TSH status in newborns from Southern Spain. Sci. Total Environ..

[49] Nagayama J., Kohno H., Kunisue T., Kataoka K., Shimomura H., Tanabe S., Konishi S. (2007). Concentrations of organochlorine pollutants in mothers who gave birth to neonates with congenital hypothyroidism. Chemosphere.

[50] Ribas-Fitó N., Sala M., Cardo E., Mazón C., de Muga M.E., Verdú A., Marco E., Grimalt J.O., Sunyer J. (2003). Organochlorine compounds and concentrations of thyroid stimulating hormone in newborns. Occup. Environ. Med..

[51] Goldner W.S., Sandler D.P., Yu F., Hoppin J.A., Kamel F., Levan T.D. (2010). Pesticide use and thyroid disease among women in the Agricultural Health Study. Am. J. Epidemiol..

[52] Boada L.D., Lara P.C., Alvarez-Leon E.E., Losada A., Zumbado M.L., Liminana-Canal J.M., Apolinario R., Serra-Majem L., Luzardo O.P. (2007). Serum levels of insulin-like growth factor-I in relation to organochlorine pesticides exposure. Growth Horm. IGF Res..

[53] Bernal J. (2007). Thyroid hormone receptors in brain development and function. Nat. Clin. Pract. Endocrinol. Metab..

[54] Gilbert M.E., Rovet J., Chen Z., Koibuchi N. (2012). Developmental thyroid hormone disruption: Prevalence, environmental contaminants and neurodevelopmental consequences. Neurotoxicology.

[55] Shelton J.F., Hertz-Picciotto I., Pessah I.N. (2012). Tipping the balance of autism risk: Potential mechanisms linking pesticides and autism. Environ. Health Perspect..

[56] Li C., Cheng Y., Tang Q., Lin S., Li Y., Hu X., Nian J., Gu H., Lu Y., Tang H. (2014). The association between prenatal exposure to organochlorine pesticides and thyroid hormone levels in newborns in Yancheng, China. Environ. Res..

[57] Yamazaki K., Araki A., Nakajima S., Miyashita C., Ikeno T., Itoh S., Minatoya M., Kobayashi S., Mizutani F., Chisaki Y. (2017). Association between prenatal exposure to organochlorine pesticides and the mental and psychomotor development of infants at ages 6 and 18 months: The Hokkaido Study on Environment and Children’s Health. Neurotoxicology.

[58] Zumbado M., Luzardo O.P., Lara P.C., Alvarez-Leon E.E., Losada A., Apolinario R., Serra-Majem L., Boada L.D. (2010). Insulin-like growth factor-I (IGF-I) serum concentrations in healthy children and adolescents: Relationship to level of contamination by DDT-derivative pesticides. Growth. Horm. IGF Res..

[59] Cools R., Nakamura K., Daw N.D. (2011). Serotonin and dopamine: Unifying affective, activational, and decision functions. Neuropsychopharmacology.

[60] ATSDR (Agency for Toxic Substances and Disease Registry) (2007). Toxicological Profile for Heptachlor and Heptachlor Epoxide.

[61] Richardson J.R., Caudle W.M., Wang M.Z., Dean E.D., Pennell K.D., Miller G.W. (2008). Developmental heptachlor exposure increases susceptibility of dopamine neurons to *N*-methyl-4-phenyl-1,2,3,6-tetrahydropyridine (MPTP)in a gender-specific manner. Neurotoxicology.

[62] Beseler C.L., Stallones L., Hoppin J.A., Alavanja M.C.R., Blair A., Keefe T., Kamel F. (2008). Depression and pesticide exposures among private pesticide applicators enrolled in the Agricultural Health Study. Environ. Health Perspect..

[63] Julvez J., Debes F., Weihe P., Choi A.L., Grandjean P. (2011). Thyroid dysfunction as a mediator of organochlorine neurotoxicity in preschool children. Environ. Health Perspect..

