# Supplementary material for: Residue Levels of Organochlorine Pesticides in Breast Milk and Its Associations with Cord Blood Thyroid Hormones and the Offspring’s Neurodevelopment
Source: Int J Environ Res Public Health. 2019 Apr 23;16(8):1438. doi: 10.3390/ijerph16081438 (PMC6517872; doi:10.3390/ijerph16081438)
Supplement: Supplementary file 1 [file ijerph-16-01438-s001.pdf]

Supplemental Materials

Residue levels of organochlorine pesticides in breast milk and its associations with cord blood thyroid hormones and the offspring's neurodevelopment

Table S1. The descriptive analysis in our participants (*n* = 55).
Table S2. Spearman's rho correlation coefficients (r) between neurodevelopmental outcomes and birth outcomes (*n* = 55).
Table S3. Spearman's rho correlation coefficients (r) between cord blood hormone levels and birth outcomes (*n* = 55).
Table S4. Spearman's rho correlation coefficients (r) between neurodevelopmental outcomes and cord-blood hormone levels (*n* = 55).
Table S5. OCP levels in breast milk in different countries.

The descriptive analysis of our participants

Table S1 shows the descriptive analysis of our participants. **A total of 32 and 23 mothers from rural and urban areas and 56.4% of their female offspring (n=31) participated in this study, respectively. Variables including working status, occupational exposure to insecticides, smoking habit, and alcohol consumption, were expressed as the frequency and percentage. The percentage of mothers who are working was 45.5% while 100% of the participants did not have any occupational exposure to OCPs. As for the smoking and drinking habits, 5.45% of the participants were reported to be smoking while 3.64% were reported to be alcohol consumers.**

Associations between birth outcomes, cord blood hormones, and Bayley-III

The correlations between the birth outcomes in our cohort, cord blood hormone levels, and Bayley-III composite scores were listed in Table S2 and S3.

Adjustment of Bayley-III scales

The 5 domains of Bayley-III scales for our participants were examined using the chronological age, which is adjusted for child's prematurity by the infant psychometrists in the standard room located in Pingtung Christian Hospital. Prior to the test of Bayley-III scales in each participant, the infant psychometrist calculated the chronological age based on instruction from the handbook of Bayley-III®. Considering the over-adjustment in examining statistical analysis, we recognized that the child's age was adjusted before the Bayley-III. The child's gestational age was not considered as the covariate in performing the statistics.

Correlations between THs and IGF-1 and Bayley-III

We examined the correlations between the neurodevelopmental outcomes and the cord-blood hormone levels. The three variables (TSH, IGF-1, and cognitive score) did not fit the normal distribution after normality was examined. The Spearman's rho correlation coefficient test was used to determine the relationships between neurodevelopmental outcomes and cord-blood hormone levels (Table S4). The result is shown below. Motor scores were positively and significantly correlated with cord blood T4, free T4, and

IGF-1, respectively. Adaptive behavioral scores had positive significant correlations with T4 and free T4 in cord blood.

Several studies have also reported the importance of thyroid ad growth hormones in the infant neurodevelopment. Thyroid deficits such as undetected hypothyroidism in pregnant mothers have been reported to cause adverse effects on the neuropsychological development of the human fetus (Haddow et al. 1999). Henrichs et al. (2010) reported that hypothyroxinemia in pregnant women was associated with delays in both expressive language and nonverbal cognitive development during early childhood. A significant risk of developing impaired psychomotor development later on in childhood has been attributed to low maternal FT4 serum concentrations (Pop et al. 1999). Severe hypothyroidism has been correlated with reduced encoding and focus in adolescents (Rovet and Hepworth 2001). According to a study by Shelton et al. (2012), brain development requires adequate thyroid hormone levels in utero. Mental and/or psychomotor deficiencies have been linked to diminished maternal T4 levels (Zimmermann 2007). **Transient intrauterine thyroid hormones deficiencies for as little as three days can alter the cortical architecture, which results in interference with neuronal migration and the growth of Purkinje cells during critical gestation stages (Roman 2007).** Both characteristic defects have been observed in autism autopsy studies conducted by Fatemi et al. (2002) and Wegiel et al. (2010). In human fetuses, sufficient thyroid hormone production only starts during week 18 of gestation (Burrow et al. 1994). Thus, sufficient maternal thyroid hormones are important in the early fetal neurodevelopment stages, most specifically for the reelin-mediated migration of neurons (Pathak et al. 2011).

The breastmilk levels of OCPs in Taiwan were within the safe criteria

The safe criteria of OCPs in breast milk were defined in the present study based on the recommendations of World Health Organization (WHO) and US Environmental Protection Agency (EPA). WHO recommended that the acceptable daily intake of DDTs was 20,000 ng/kg/day for a breastfed infant (WHO, 1986). The website of US EPA Integrated Risk Information System (IRIS) also provided a reference dose for oral exposure (RfD) for several OCP compounds for example: the RfD of DDT is 5x10-4 mg/kg/day (US EPA, 2019). The estimated median DDTs daily intake of 91.2 ng/kg/day for an exclusively breastfed infant was calculated based on assumption that a breastfed infant weighted 4 kg and consumed 699 g milk/day in the first month after delivery in the present study. Our values were extremely lower compared with WHO recommendation and RfDs listed in US EPA IRIS. We also listed several RfDs of OCPs from the IRIS website as the follows: methoxychlor of 5x10-3 and dieldrin of 5x10-5.

References

US EPA Integrated Risk Information System https://www.epa.gov/iris (Access Mar 30 2019)

WHO, 1986. Principles for evaluating health risks from chemicals during infancy and early childhood: the need for a special approach. Environmental Health Criteria, 59. WHO, Geneva.

Breast milk levels of OCPs in different countries

The breastmilk levels of OCPs in Taiwan were within the safe criteria. Our OCP levels detected in the breast milk samples collected between 2007 and 2010 were observed to be lower than the reports from Korea (Lee et al. 2013), Vietnam (Minh et al. 2004), China (Zhou et al. 2012), and Japan (Haraguchi et al. 2009), and Russia (Mamontova et al. 2017) with comparable sampling years. Breastmilk OCP levels in our research were similar magnitudes with the Chinese study (Zhou et al. 2012) (Table S5).

Table S1. The descriptive analysis in our participants.

Variables	Frequency (number)	Percentage (%)
Residence area		
Urban area	23	43.6
Rural area	32	56.4
Working status and type		
No job	25	45.5
Having job	30	54.5
Teachers and employees in school	9	30.0
Medical staff	6	20.0
Employees in factory	4	13.3
Employees in service industry	5	16.7
Employees in business	3	10.0
Others	3	10.0
Occupational exposure of insecticides		
NO	55	100
Yes	0	0
Smoking habit		
NO	52	94.5
Yes	3	5.45
Drinking habit		
NO	53	96.4
Yes	2	3.64

Table S2 Spearman's rho correlation coefficients (r) between neurodevelopmental outcomes and birth outcomes (n=55).

	Infant's birth outcomes			
Bayley-III	Birth weight	Birth length	Head circumference	Chest circumference
Cognitive	0.178	0.151	-0.163	0.077
Language	0.179	0.089	0.093	0.208
Motor	0.342**	0.535	0.113	0.209
Social emotional	0.168	0.265*	0.126	-0.024
Adaptive behavioral	0.294*	0.396**	0.057	0.132

*p<0.05, **p<0.01, ***p<0.001.

Table S3. Spearman's rho correlation coefficients (r) between cord blood hormone levels and birth outcomes (n=55).

	Infant's birth outcomes			
Bayley-III	Birth weight	Birth length	Head circumference	Chest circumference
Cord blood T3	-0.152	-0.130	-0.216	-0.188
Cord blood T4	0.188	0.095	0.183	0.094
Cord blood TSH	-0.123	-0.008	-0.014	-0.135
Cord blood FT3	-0.111	-0.017	-0.117	-0.040
Cord blood FT4	0.335*	0.290*	0.239	0.164

Cord blood IGF-1	0.160	0.175	0.136	-0.023

*$p<0.05$, **$p<0.01$, ***$p<0.001$.

Table S4 Spearman's rho correlation coefficients (r) between neurodevelopmental outcomes and cord-blood hormone levels (n=55).

Bayley-III	Cord blood hormones					
	T3	T4	TSH	Free T3	Free T4	IGF-1
Cognitive	-0.020	0.023	0.151	-0.102	0.061	0.079
Language	-0.014	0.007	0.010	0.004	0.158	-0.079
Motor	-0.042	0.270*	-0.07	0.039	0.271*	0.383**
Social emotional	-0.107	0.220	0.146	0.110	0.248	0.141
Adaptive behavioral	0.184	0.298*	0.119	0.158	0.274*	0.148

*$p<0.05$, **$p<0.01$, ***$p<0.001$.

Table S5. OCP levels in breast milk in different countries.

Country	Calendar Period	Measured Compound	Pesticide Level	Reference
Taiwan	2000–2001	Aldrin	<LOD	Chao et al. (2006)
		Dieldrin	<LOD	
		Endrin	<LOD	
		β-HCH	1.2 ng/g lipid	
		γ-HCH	0.8 ng/g lipid	
		α-CHL	7.4 ng/g lipid	
		Heptachlor	2.3 ng/g lipid	
		Heptachlor epoxide	4.0 ng/g lipid	
		p,p′-DDT	19 ng/g lipid	
		p,p′-DDE	228 ng/g lipid	
Thailand	1998	Heptachlor	4.3 ng/mL	Stuetz et al. (2001)
		Heptachlor epoxide	4.4 ng/mL	
		γ-HCH	3.6 ng/mL	
		p,p′-DDT	69.4 ng/mL	
		p,p′-DDE	169.4 ng/mL	
		p,p′-DDD	6.8 ng/mL	
China	1999–2000	p,p′-DDT	0.70 μg /g fat	Wong et al. (2002)
		p,p′-DDE	2.85 μg/ g fat	
		β-HCH	1.11 μg/g fat	
China	2003–2005	α-HCH	76.16 ng/g lipid	Zhao et al. (2007)
		γ-HCH	16.67 ng/g lipid	
		β-HCH	214.33 ng/g lipid	
		p,p′-DDE	1528.20 ng/g lipid	
China	2006, 2008, 2010	α-HCH	<LOD	Zhou et al. (2012)
		β-HCH	67.1 ng/g lipid	
		γ-HCH	<LOD	
		δ-HCH	>LOD	
		HCB	25.5 ng/g lipid	
		2,4′-DDE	<LOD	

		4,4′-DDE	<LOD	
		2,4′-DDD	<LOD	
		4,4′-DDD	10.5 ng/g lipid	
Vietnam	2000–2001	p,p′-DDT	34–6900 ng/g lipid wt	Minh et al. (2004)
		p,p′-DDE	420–6300 ng/g lipid wt	
		p,p′-DDD	3–50 ng/g lipid wt	
		β-HCH	11–160 ng/g lipid wt	
Korea	2011	p,p′-DDT	91.7 ng/g lipid wt	Lee et al. (2013)
		p,p′-DDE	0.94 ng/g lipid wt	
		p,p′-DDD	6.51 ng/g lipid wt	
		β-HCH	20.5 ng/g lipid wt	
		Heptachlor epoxide	2.22 ng/g lipid wt	
USA, MEXICO and RUSSIA	1999, 2002, 2007, 2009, 2011	HCB	0.80–3.00 ng/g lipid wt	Mannetje et al. (2012)
		β-HCH	0.51–2.57 ng/g lipid wt	
		p,p′-DDT	0.42–1.41 ng/g lipid wt	
		p,p′-DDE	0.56–1.40 ng/g lipid wt	
USA	2004	p,p′-DDT	<0.6 ng/g lipid wt	Johnson-Restrepo et al. (2007)
		p,p′-DDE	35.3ng/lipid wt	
		p,p′-DDD	2.7 ng/lipid wt	
		α-HCH	1.4 ng/g lipid wt	
		β-HCH	4.4 ng/g lipid wt	
		γ-HCH	5.1 ng/g lipid wt	
		δ-HCH	<1.6 ng/g lipid wt	
Russia	1997–2009	HCB	29 ng/g lipid	Mamontova et al. (2017)
		α-HCH	3.1 ng/g lipid	
		γ-HCH	0.56 ng/g lipid	
		p,p′-DDT	32 ng/g lipid	
		p,p′-DDE	491ng/lipid	
		p,p′-DDD	1.9 ng/lipid	
Colombia	Unspecified	4,4′ DDE	126 ng/g lipid wt	Rojas-Squella et al. (2013)
		4,4′ DDE	203 ng/g lipid wt	
Norway	2002–2006	p,p′-DDE	41 ng/g lipid wt	Polder et al. (2009)
		HCB	11ng/g lipid wt	
		β-HCH	4.7 ng/g lipid wt	
		Oxychlordane	2.8 ng/g lipid wt	
Vietnam, China, and Japan	2007–2008	p,p′-DDT	5.8 ng/g lipid	Haraguchi et al. (2009)
		p,p′-DDE	160 ng/lipid	
		p,p′-DDD	1.4 ng/lipid	
		o,p′-DDT	0.84 ng/g lipid	
		Oxychlordane	3.7 ng/g lipid	
		β-HCH	140 ng/g lipid	
		HCB	13 ng/g lipid	

MDL: method detection limit. [a] ΣHCH is the sum of α, β, γ, and δ- HCH; ΣCHL is the sum of cis- and trans-CHL; ΣDDT is the sum of 4,4′-DDD, 4,4′-DDE, and 4,4′-DDT; ΣEndosulfan is the sum of endosulfanI, endosulfan II, and endosulfan sulfate; ΣEndrin is the sum of endrin, endrinaldehyde, and endrinketone; ΣHeptachlor is the sum of heptachlor and heptachlor epoxide.

References

1. Burrow, G.N., Fisher, D.A. and Larsen, P.R. (1994) Maternal and fetal thyroid function. N Engl J Med 331(16), 1072-1078.
2. Chao, H.R., Wang, S.L., Lin, T.C. and Chung, X.H. (2006) Levels of organochlorine pesticides in human milk from central Taiwan. Chemosphere 62(11), 1774-1785.
3. Fatemi, S.H., Halt, A.R., Realmuto, G., Earle, J., Kist, D.A., Thuras, P. and Merz, A. (2002) Purkinje cell size is reduced in cerebellum of patients with autism. Cell Mol Neurobiol 22(2), 171-175.
4. Haddow, J.E., Palomaki, G.E., Allan, W.C., Williams, J.R., Knight, G.J., Gagnon, J., O'Heir, C.E., Mitchell, M.L., Hermos, R.J., Waisbren, S.E., Faix, J.D. and Klein, R.Z. (1999) Maternal thyroid deficiency during pregnancy and subsequent neuropsychological development of the child. N Engl J Med 341(8), 549-555.
5. Haraguchi, K., Koizumi, A., Inoue, K., Harada, K.H., Hitomi, T., Minata, M., Tanabe, M., Kato, Y., Nishimura, E., Yamamoto, Y., Watanabe, T., Takenaka, K., Uehara, S., Yang, H.R., Kim, M.Y., Moon, C.S., Kim, H.S., Wang, P., Liu, A. and Hung, N.N. (2009) Levels and regional trends of persistent organochlorines and polybrominated diphenyl ethers in Asian breast milk demonstrate POPs signatures unique to individual countries. Environ Int 35(7), 1072-1079.
6. Henrichs, J., Bongers-Schokking, J.J., Schenk, J.J., Ghassabian, A., Schmidt, H.G., Visser, T.J., Hooijkaas, H., de Muinck Keizer-Schrama, S.M., Hofman, A., Jaddoe, V.V., Visser, W., Steegers, E.A., Verhulst, F.C., de Rijke, Y.B. and Tiemeier, H. (2010) Maternal thyroid function during early pregnancy and cognitive functioning in early childhood: the generation R study. J Clin Endocrinol Metab 95(9), 4227-4234.
7. Johnson-Restrepo, B., Addink, R., Wong, C., Arcaro, K. and Kannan, K. (2007) Polybrominated diphenyl ethers and organochlorine pesticides in human breast milk from Massachusetts, USA. J Environ Monit 9(11), 1205-1212.
8. Lee, S., Kim, S., Lee, H.-K., Lee, I.-S., Park, J., Kim, H.-J., Lee, J.J., Choi, G., Choi, S., Kim, S., Kim, S.Y., Choi, K., Kim, S. and Moon, H.-B. (2013) Contamination of polychlorinated biphenyls and organochlorine pesticides in breast milk in Korea: Time-course variation, influencing factors, and exposure assessment. Chemosphere 93(8), 1578-1585.
9. Mamontova, E.A., Tarasova, E.N. and Mamontov, A.A. (2017) PCBs and OCPs in human milk in Eastern Siberia, Russia: Levels, temporal trends and infant exposure assessment. Chemosphere 178, 239-248.
10. Mannetje, A., Coakley, J., Mueller, J.F., Harden, F., Toms, L.M. and Douwes, J. (2012) Partitioning of persistent organic pollutants (POPs) between human serum and breast milk: a literature review. Chemosphere 89(8), 911-918.
11. Minh, N.H., Someya, M., Minh, T.B., Kunisue, T., Iwata, H., Watanabe, M., Tanabe, S., Viet, P.H. and Tuyen, B.C. (2004) Persistent organochlorine residues in human breast milk from Hanoi and Hochiminh City, Vietnam: contamination, accumulation kinetics and risk assessment for infants. Environmental Pollution 129(3), 431-441.
12. Pathak, A., Sinha, R.A., Mohan, V., Mitra, K. and Godbole, M.M. (2011) Maternal thyroid hormone before the onset of fetal thyroid function regulates reelin and downstream signaling cascade affecting neocortical neuronal migration. Cereb Cortex 21(1), 11-21.
13. Polder, A., Skaare, J.U., Skjerve, E., Loken, K.B. and Eggesbo, M. (2009) Levels of chlorinated pesticides and polychlorinated biphenyls in Norwegian breast milk (2002-2006), and factors that may predict the level of contamination. Sci Total Environ 407(16), 4584-4590.
14. Pop, V.J., Kuijpens, J.L., van Baar, A.L., Verkerk, G., van Son, M.M., de Vijlder, J.J., Vulsma, T., Wiersinga, W.M., Drexhage, H.A. and Vader, H.L. (1999) Low maternal free thyroxine concentrations during early pregnancy are associated with impaired psychomotor development in infancy. Clin Endocrinol (Oxf) 50(2), 149-155.
15. Rojas-Squella, X., Santos, L., Baumann, W., Landaeta, D., Jaimes, A., Correa, J.C., Sarmiento, O.L. and Ramos-Bonilla, J.P. (2013) Presence of organochlorine pesticides in breast milk samples from Colombian women. Chemosphere 91(6), 733-739.
16. Roman, G.C. (2007) Autism: transient in utero hypothyroxinemia related to maternal flavonoid ingestion during pregnancy and to other environmental antithyroid agents. J Neurol Sci 262(1-2), 15-26.
17. Rovet, J.F. and Hepworth, S. (2001) Attention problems in adolescents with congenital hypothyroidism: a multicomponential analysis. J Int Neuropsychol Soc 7(6), 734-744.

18. Shelton, J.F., Hertz-Picciotto, I. and Pessah, I.N. (2012) Tipping the balance of autism risk: potential mechanisms linking pesticides and autism. Environmental health perspectives 120(7), 944-951.
19. Stuetz, W., Prapamontol, T., Erhardt, J.G. and Classen, H.G. (2001) Organochlorine pesticide residues in human milk of a Hmong hill tribe living in Northern Thailand. Sci Total Environ 273(1-3), 53-60.
20. Wegiel, J., Kuchna, I., Nowicki, K., Imaki, H., Wegiel, J., Marchi, E., Ma, S.Y., Chauhan, A., Chauhan, V., Bobrowicz, T.W., de Leon, M., Louis, L.A., Cohen, I.L., London, E., Brown, W.T. and Wisniewski, T. (2010) The neuropathology of autism: defects of neurogenesis and neuronal migration, and dysplastic changes. Acta Neuropathol 119(6), 755-770.
21. Wong, C.K., Leung, K.M., Poon, B.H., Lan, C.Y. and Wong, M.H. (2002) Organochlorine hydrocarbons in human breast milk collected in Hong Kong and Guangzhou. Arch Environ Contam Toxicol 43(3), 364-372.
22. Zhao, G., Xu, Y., Li, W., Han, G. and Ling, B. (2007) PCBs and OCPs in human milk and selected foods from Luqiao and Pingqiao in Zhejiang, China. Sci Total Environ 378(3), 281-292.
23. Zhou, J., Zeng, X., Zheng, K., Zhu, X., Ma, L., Xu, Q., Zhang, X., Yu, Y., Sheng, G. and Fu, J. (2012) Musks and organochlorine pesticides in breast milk from Shanghai, China: levels, temporal trends and exposure assessment. Ecotoxicol Environ Saf 84, 325-333.
24. Zimmermann, M.B. (2007) The adverse effects of mild-to-moderate iodine deficiency during pregnancy and childhood: a review. Thyroid 17(9), 829-835.
